# Uric Acid Enhances Neurogenesis in a Parkinsonian Model by Remodeling Mitochondria

**DOI:** 10.3389/fnagi.2022.851711

**Published:** 2022-06-02

**Authors:** Ji Eun Lee, Yu Jin Shin, Yi Seul Kim, Ha Na Kim, Dong Yeol Kim, Seok Jong Chung, Han Soo Yoo, Jin Young Shin, Phil Hyu Lee

**Affiliations:** ^1^Department of Neurology, Yonsei University College of Medicine, Seoul, South Korea; ^2^Department of Neurology, Yongin Severance Hospital, Yonsei University Health System, Yongin, South Korea; ^3^Severance Biomedical Science Institute, Yonsei University, Seoul, South Korea

**Keywords:** uric acid, neurogenesis, Parkinson’s disease, mitochondrial dynamics, neural precursor cell

## Abstract

**Background:**

Adult neurogenesis is the process of generating new neurons to enter neural circuits and differentiate into functional neurons. However, it is significantly reduced in Parkinson’s disease (PD). Uric acid (UA), a natural antioxidant, has neuroprotective properties in patients with PD. This study aimed to investigate whether UA would enhance neurogenesis in PD.

**Methods:**

We evaluated whether elevating serum UA levels in a 1-methyl-4-phenyl-1,2,3,6-tetrahydropyridine (MPTP)-induced parkinsonian mouse model would restore neurogenesis in the subventricular zone (SVZ). For a cellular model, we primary cultured neural precursor cells (NPCs) from post-natal day 1 rat and evaluated whether UA treatment promoted cell proliferation against 1-methyl-4-phenylpyridinium (MPP^+^).

**Results:**

Uric acid enhanced neurogenesis in both *in vivo* and *in vitro* parkinsonian model. UA-elevating therapy significantly increased the number of bromodeoxyuridine (BrdU)-positive cells in the SVZ of PD animals as compared to PD mice with normal UA levels. In a cellular model, UA treatment increased the expression of Ki-67. In the process of modulating neurogenesis, UA elevation up-regulated the expression of mitochondrial fusion markers.

**Conclusion:**

In MPTP-induced parkinsonian model, UA probably enhanced neurogenesis *via* regulating mitochondrial dynamics, promoting fusion machinery, and inhibiting fission process.

## Introduction

Neurogenesis is the ability of the brain to produce neurons and strengthen existing connections between neuronal cells across the lifespan. Remarkably, the brain continually generates new neurons even after embryonic development. Under normal physiology, new neurons are generated from neural stem cells (NSCs) of the subventricular zone (SVZ) or subgranular zone, which then migrate to the neurogenesis niche, enter neural circuits, and differentiate into functional neurons (Berdugo-Vega et al., 2020). This process is known to be involved in various brain functions such as memory formation, motor control, neuronal plasticity, and endogenous recovery. Ample evidence has demonstrated that patients with neurodegenerative diseases such as Alzheimer’s disease, Parkinson’s disease (PD), and Huntington’s disease show deficient neurogenesis compared to healthy controls (Gil-Mohapel et al., 2011; Winner and Winkler, 2015; Scopa et al., 2020). Gradual loss of neuronal populations and diseased neurons disrupt synaptic transmission, cell renewal, and putative function in neurodegenerative diseases (Luo and O’Leary, 2005). Specifically, SVZ is under control of dopaminergic afferents from the substantia nigra (SN) (Hoglinger et al., 2004). In parkinsonian environment, dopaminergic deafferentation results in decreased proliferation in SVZ, reducing population of neural stem cell which will differentiate into dopaminergic neurons (Marxreiter et al., 2013). Thus, restoring adult neurogenesis is considered to be one of the important strategies for treating patients suffering from neurodegenerative diseases (Geraerts et al., 2007).

Parkinson’s disease is characterized by the selective loss of dopaminergic neurons in the (SN) and the degeneration of projecting nerve fibers to the striatum, leading to parkinsonian motor symptoms such as bradykinesia, rigidity, and tremor (Jankovic, 2008). Among the multiple factors underlying PD pathogenesis, oxidative stress plays a key role in PD pathology *via* disrupting the electron transport chain and subsequent electron leakage from donor redox to molecular oxygen (Hwang, 2013). Moreover, oxidative stress is intertwined with other mechanisms implicated in PD, including protein misfolding and aggregation, mitochondrial dysfunction, and apoptosis (Picca et al., 2020). Uric acid (UA), purine metabolite, is a powerful antioxidant, present intracellularly and in all body fluids. It not only scavenges reactive oxygen species (ROS) but also blocks the reaction of superoxide anion with nitric oxide that can injure cells by nitrosylating the tyrosine residues of proteins, and prevents extracellular superoxide dismutase degradation (Squadrito et al., 2000). Several epidemiological studies reported that UA has neuroprotective properties against PD, showing that PD patients with higher UA have reduced risk of PD incidence as well as slower disease progression (Wen et al., 2017; Tana et al., 2018). Additionally, the beneficial effects of UA have been observed in other neurodegenerative diseases, such as amyotrophic lateral sclerosis (Bakshi et al., 2018), Alzheimer’s disease (Scheepers et al., 2019), Huntington’s disease (Auinger et al., 2010), or other disorders (Li et al., 2018; Ya et al., 2018). These studies imply that in addition to antioxidant properties, UA may have another pathway to exerting neuroprotective effects against neurodegenerative conditions.

Emerging evidence indicates that mitochondria are central regulators of NSC fate decisions and are crucial for adult neurogenesis (Beckervordersandforth, 2017). NSCs are not only dependent on generation of mitochondrial metabolites (Zheng et al., 2018), but are also dependent on changes in mitochondrial morphology and metabolic properties across various stages of differentiation (Khacho et al., 2016). Mitochondrial malfunction makes stem cells vulnerable to oxidative stress, which in turn accelerates NSC death, resulting in reduced neurogenesis (Khacho et al., 2017). As mitochondria are dynamic organelles that change their size and morphology actively, the processes of fission and fusion oppose each other and allow the mitochondria to constantly remodel themselves depending on their environment (Hollenbeck and Saxton, 2005; Okamoto and Shaw, 2005). A recent study found that adult neurogenesis in the hippocampus is critically dependent on mitochondrial complex function in mice and NSCs isolated from mice with malfunctioning mitochondria (Beckervordersandforth et al., 2017). Indeed, mitochondrial dynamics may have an important role in neurogenesis *via* maintaining a functional mitochondrial network during biogenesis (Flippo and Strack, 2017; Khacho and Slack, 2018; Arrázola et al., 2019). In this study, we hypothesized that UA would enhance neurogenesis by controlling mitochondrial dynamics in PD. To do this, we evaluated whether high levels of UA increase neurogenic activity in primary cultured neural precursor cells (NPCs) and SVZ of 1-methyl-4-phenyl-1,2,3,6-tetrahydropyridine (MPTP)-treated animals. In addition, we examined possible role of mitochondrial dynamics in UA-mediated modulation of neurogenesis in PD models.

## Materials and Methods

### Parkinsonian Animal Model and Drugs Administration

All procedures were performed in accordance with the Laboratory Animals Welfare Act, the Guide for the Care and Use of Laboratory Animals and the Guidelines and Policies. The rodent experiment was approved by IACUC (Institutional Animal Care and Use Committee) in the Yonsei University Health System. ARRIVE guidelines were followed.^1^ Male C57BL/6J mice (4 weeks old) were acclimated in a climate-controlled room with a constant 12 h light/dark cycle (12 h on, 12 h off) for a week prior to the initiation of drug administration. At 5 weeks of age, the mice were randomly divided into three groups: control group, MPTP PD group, and MPTP PD + UA treatment group. To elevate serum UA levels, the UA treatment group received an i.p injection of KOx (Sigma, 500 mg/kg) and IMP (Sigma, 500 mg/kg) daily for 2 weeks while others received normal saline. To construct parkinsonian model, the mice (except for those in the control group) received a sub-acute injection of MPTP freshly dissolved in 20% DMSO/80% normal saline (25 mg/kg) by i.p injection (Sigma, St. Louis, MO, United States) for 5 days. After 72 h had passed since the last MPTP injection, the UA groups received 4 weeks of UA injections. During drug administration period, the control group mice received same volume of saline i.p injection. Four days prior to last UA injection, all animals received a bromodeoxyuridine (BrdU) injection (Sigma, 100 mg/kg) daily on five consecutive days.

### Preparation of Serum and Brain Tissue

At the end of the experimental period, mice were deeply anesthetized with isoflurane and their blood and brains were collected. Mouse blood was collected from the abdominal aorta. To evaluate whether serum UA levels were elevated by injection of KOx and IMP, mouse blood samples were collected in SST tubes (BD Diagnostic Systems, Sparks, MD, United States), and serum and blood cells were separated by centrifugation (2000 × *g* for 20 min). The isolated serum samples were rapidly frozen and stored at −20°C until analyzed. For immunohistochemistry, the mice were perfused with 4% paraformaldehyde. Brains were harvested from the skulls, post-fixed overnight in 4% paraformaldehyde, and stored in 30% sucrose solution for 1–2 days at 4°C until they sank. Finally, 25-μm coronal sections were obtained using cryostat. The sections were stored in tissue storage solution (30% glycerol, 30% ethylene glycol, 30% distilled water, 10% 0.2 M PB) at 4°C until required (Na Kim et al., 2017).

### Neural Precursor Cell Primary Culture and Identification

Neural precursor cells were isolated from a postnatal day-1 Sprague-Dawley rat. Offspring of rats were decapitated under halothane anesthesia. Brain tissue was taken from the rat and a wedge of tissue was microdissected from the portion of the lateral ventricle that included the anterior part of the SVZ. The tissues were incubated in Hank’s balanced salt solution (HBSS) for 10 min and trypsin for 3 min at room temperature. NPCs were dissociated into single cells by pipetting, and Dulbecco’s modified eagle medium (DMEM) supplementation with 10% fetal bovine serum (FBS; Hyclone) and 1% penicillin/streptomycin (P/S; Hyclone) was added to inhibit trypsin function. Cells were plated in 100-mm plastic culture dishes and cultivated in low glucose DMEM in a humidified incubator at 37°C under 5% CO_2_. After 24 h, non-adherent cells were removed by changing media. To identify characteristics of NPCs, total RNA was extracted from the NPCs using Trizol reagent (Lugen Sci, Korea) according to the manufacturer’s instructions. RNA concentration was measured by absorption at 260 nm using NanoDrop Lite Spectrophotometer (Thermo Scientific, Wilmington, DE, United States) and an equal amount of RNA (approximately 1 ug) in each experiment was reverse transcribed. The PCR reaction was performed using 10 pmol each of the primers for NES (forward 5′-GGCCACAGTGCCTAGTTCTT-3′, reverse 5′-GTTCCCAGATTTGCCCCTCA), SOX2 (forward 5′-TAAGTACACGCTTCCCGGAG-3′, reverse 5′-CATCATGC TGTAGCTGCCGT-3′), DCX (forward 5′-TCACAGCATCT CCACCCAAC-3′, reverse 5′-ATGCCTGCAAGGTTCTGGTT-3′), MSI1 (forward 5′-CGGAGAGCACAGCCTAAGAT-3′, reverse 5′-TCGAACGTGACAAACCCGAA-3′), and GFAP (forward 5′-ACGAGGCTAATGACTATCGC-3′, reverse 5′-GTT TCTCGGATCTGGAGGTT-3′). After an initial denaturation at 95°C for 10 min, 30 cycles of PCR were performed, consisting of denaturation at 95°C for 20 s, annealing at 60°C for 30 s, extension at 72°C for 60 s followed by a final extension at 72°C for 5 min. The PCR products were separated by electrophoresis on 1.5% agarose gel and stained with Noble View (Noble Bio). Gels were examined under UV illumination Gel doc (MiniBISpro, DNR).

### Neural Precursor Cell Treatment

Identified NPCs (positive for NES, sox2, DCX, and MSI1, but negative for GFAP and CD11b) were seeded in 96-well cell culture plates (SPL) at a density of 0.5 × 10^4^/well. Plates were incubated at 37°C for 72 h to allow cells to be attached. Solution of MPP^+^ and UA were prepared in advance. MPP^+^ (sigma) was dissolved in distilled water to a final concentration of 100 mM stock. After stabilization, each well was randomly divided into three groups as follows: control group, MPP^+^ group, MPP^+^/UA group. MPP^+^/UA group cells were treated with 150 μM of UA while others were just changed media. After 24 h, the MPP^+^ group was treated with 150 μM of MPP^+^ and the UA group was treated with 150 μM of MPP^+^ and UA mixture. Plates were incubated in a cell incubator for 72 h. 150 μM of MPP^+^ was treated to the MPP^+^ group cells and the MPP^+^ and UA mixture were treated to both UA groups. Plates were incubated in a cell incubator for an additional 72 h.

### Cell Viability Analysis

Cell viability was measured by MTS cell proliferation assay (promega). According to the assay manufacturer’s instructions, right before the measurement time, MTS and PMS were mixed in a 1 ml:50 μl ratio and incubated for 10 min. Then, 20 μl of mix was added to each well. The plates were incubated at 37°C for 1 h, and we measured the absorbance at 490 nm using a 96-well plate reader. Cell viability values were expressed as experimental group/average of control group × 100 = viability percentage. All experiments were repeated at least three times.

### Measurements of Serum Uric Acid Level

Serum UA levels were measured using an Amplex Red Uric Acid/Uricase Assay Kit (Molecular Probes, Eugene, OR, United States) according to the manufacturer’s instructions. Each serum samples were 1/10 diluted for measurement. Briefly, uricase catalyzes the conversion of UA to allantoin, H_2_O_2_, and CO_2_. Under the presence of horseradish peroxidase, H_2_O_2_ reacts stochiometrically with Amplex Red reagent to generate the red-fluorescent oxidation product, resorufin, which is measured using excitation at 540 nm and detection at 590 nm.

### Measurements of Reactive Oxygen Species Level

The level of intracellular ROS in NPCs was measured using 2′,7′-Dichlorofluorescin diacetate (DCFDA), DCFDA Cellular ROS Detection Assay Kit (ab113851, Abcam, United Kingdom). This reagent diffuses into the cell, which goes through deacetylation and oxidization by cellular enzymes into 2′,7′-dichlorofluorescein (DCF). According to manufacturer’s protocol, cells were seeded at 1 × 10^4^ cells/well in 96-well plate. When chemical treatment was done, cells were washed twice with 1X buffer. Washed cells were incubated with 25 μM DCFDA solution for 45 min at 37°C. Then, DCFDA solution was removed, cells were washed with buffer again, and the level of ROS was measured using excitation at 485 nm and detection at 535 nm.

### Immunohistochemistry

When drug administration was done in the animal model, brain tissue was immunostained with anti-BrdU and TH antibodies (1:500, Sigma, St. Louis, MO, United States) in SVZ and SN respectively to investigate newborn neurons and dopaminergic neurons, which we interpret as the degree of neurogenesis and as confirmation of the MPTP parkinsonian model. First, brain tissues were frozen with O.C.T. compound (Sakura Finetek) and 25-um coronal sections were obtained using cryostat. Sections were immunostained using immunofluorescence analysis or 3,3-diaminobenzidine (DAB) method. For BrdU detection, the slides were washed three times in PBS, incubated with 50% formamide in 2X SSC DW (standard saline citrate distilled water) for 2 h at 65°C for antigen retrieval, and rinsed three times in 2X SSC DW. After rinsing, the slides were incubated with 2 N HCl in DW for 30 min at 37°C to denature DNA and rinsed three times in PBS. They were incubated in borate buffer (0.1 M, pH-8.5) to neutralize the acidic medium and then blocked in 3% H_2_O_2_ to remove endogenous peroxidase for 10 min. To reduce non-specific binding, slides were blocked with 1% BSA in PBST for 2 h at room temperature and incubated with primary antibody, mouse anti-BrdU (1:500, Thermofisher Scientific, Waltham, MA, United States) for overnight at 4°C. As secondary antibodies, 1:200 dilutions of biotinylated in blocking solution which was visualized with 0.05% diaminobenzidine (DAB, Dako, Carpinteria, CA, United States). Immunofluorescence labeling was carried out by incubating tissue slides in donkey anti-mouse IgG and goat anti-rabbit IgG (both Alexa Fluor-488, green and Alexa Fluor-555, red) secondary antibodies (1:200, invitrogen). The cell nuclei were counterstained with 4′,6-diamidino-2-phenylindole (DAPI; 1:2000 dilution, invitrogen). The brain tissue was dried and stained with Mayer’s hematoxylin (Muto Pure Chemicals Ltd., Tokyo, Japan). The immunostained tissues were analyzed using bright-field microscopy and immunofluorescence images were viewed with a Zeiss LSM 700 confocal microscope (Carl Zeiss, Berlin, Germany) (Oh et al., 2015).

### Quantitative Real-Time PCR

Total RNA was isolated from NPCs using Trizol reagent (Lugen Scei, Korea) according to manufacturer’s instructions. An equal amount of RNA (approximately 1 ug) in each experiment was reverse transcribed using an cDNA synthesis premix (Applied Biosystems, Promega, Madison, WI, United States). A master mix of the following reaction components was prepared to the indicated end-concentration: 12.5 μl of 2X SYBR green buffer (Promega, United States), 2 μl of forward and reverse primer (10 pM), and 2 μl DNA template (100 ng). Amplification conditions were as follows: initial denaturation at 95°C for 2 min, followed by 40 amplification cycles of 95°C for 15 s and 60°C for 1 min to anneal and extend, respectively. Quantitative PCR experiments were performed using Applied Biosystems (Thermofisher Scientific, Waltham, MA, United States). The quantitative real-time PCR reaction was performed using 10 pmol each of the primers for rat PGC1-α (forward 5′-GGACATGTGCAGCCAAGACT-3′, reverse 5′-TCGAATATGTTCGCGGGCTC), FIS1 (forward 5′-CGTGCTTTCTGTAACGCCTG-3′, reverse 5′-CTACAGGCA CTTTGGGGGTT-3′), MFN2 (forward 5′-AAGAGCTCAG GGGACGGTAT-3′, reverse 5′-GCAAGGTGAGCCTTACAG GT-3′), and TFAM (forward 5′-GTGATCTCATCCGTCG CAGT-3′, reverse 5′-CATTCAGTGGGCAGAAGTCCA-3′) (Kim et al., 2021).

### MitoTracker Staining

To visualize mitochondria, MitoTrackerRed CMXRos (Invitrogen, Camarillo, CA, United States) was diluted in NPC cell culture media to a final concentration of 50 nM according to manufacturer’s instructions. The cells were incubated under normal culture conditions for 30 min, fixed with 1:1 ratio of methanol and acetone for 5 min at −20°C, and permeabilized with 0.1% triton X-100 in PBS. All NPCs were counter stained for nuclei using DAPI (blue), and then visualized by a Zeiss LSM 700 confocal microscope (Carl Zeiss, Berlin, Germany) and Zen imaging software. All cells were imaged using identical exposure times and laser power settings.

### Electron Microscope Analysis

Primary cultured NPCs were fixed with 2% glutaraldehyde - paraformaldehyde in 0.1 M PB (pH 7.4) for 12 h and then washed twice for 30 min in 0.1 M PB. They were post-fixed with 1% OsO4 dissolved in 0.1 M PB for 2 h and dehydrated in an ascending gradual series (50–100%) of ethanol and infiltrated with propylene oxide. Specimens were embedded using a Poly/Bed 812 kit (Polysciences). After pure fresh resin embedding and polymerization at 65°C in an electron microscope oven (TD-700, DOSAKA) for 24 h, 200 nm-thick sections were initially cut and stained with toluidine blue for light microscopy. Thin sections (80 nm) were double stained with 3% uranyl acetate and lead citrate for contrast staining. The sections were cut using a Leica electron microscope (EM) UC7 Ultra-microtome (Leica Microsystems). All of the thin sections were observed by transmission electron microscopy (TEM) (JEM-1011, JEOL) at an acceleration voltage of 80 kV.

### Mitochondrial Morphology Assessment

To quantify mitochondrial morphology, mitochondrial tubule length was measured. Mitochondrial tubule of 5 μm or more was determined as tubular. Mitochondria tubule between 0.5 and 5 μm but none more than 5 μm was determined as intermediate. None of more than 0.5 μm determined as fragmented. If more than 75% mitochondria were tubular a cell was judged to have tubular form. If cells presented mitochondria with mixed morphologies, they were classified as intermediate. If none of mitochondria were more than 0.5 μm, they were determined as fragmented (Duvezin-Caubet et al., 2006). For the TEM analysis, mitochondria with intact membrane and matrix were considered as normal while mitochondria with broken membrane and abnormal morphology with large vacuole area (over 20% of the total organelle) were determined as swollen (Xiong et al., 2016). We also analyzed mitochondrial network using Mitochondrial Network Analysis (MiNA) toolset. Mitotracker images were pre-processed using unsharp mask and skeletonized to visualize mitochondrial wireframe using Image J. The skeletonized feature was measured and analyzed by MiNA (Valente et al., 2017).

### Western Blotting Analysis

When animal drug administration was over, five animals in each group were fixed with paraformaldehyde while other five animals’ brain tissue was dissected under a microscope. The portion of the lateral ventricle that included the SVZ was dissected and dissolved in ice-cold RIPA buffer (50 mM Tris-HCl, pH 7.5, with 150 mM sodium chloride, 1% triton X-100, 1% sodium deoxycholate, 0.1% SDS, 2 mM EDTA sterile solution; Lugen Sci, Korea) plus protease inhibitor cocktail (Sigma). The lysates were centrifuged at 4°C for 20 min (14,000 g), and supernatants were transferred to fresh tubes. Briefly, 30 ug of protein was separated by SDS-gel electrophoresis and transferred to hydrophobic polyvinylidene difluoride (PVDF) membranes (GE Healthcare, Little Chalfont, United Kingdom). DLP1 analysis was performed in non-reducing condition. The membranes were blocked in 5% skim milk in PBST. Membranes were probed with the following primary antibodies: mouse anti-MFN1 (Abcam), rabbit anti-MFN2 (Epitomics), mouse anti-OPA1 (BD biosciences), rabbit anti-FIS1 (Abnova), mouse anti-Dlp1 (BD biosciences), mouse anti-NES (Millipore), and mouse anti-Actin (santacruz). As secondary antibodies, 1:5000 dilutions of horeseradish peroxidase-conjugated goat anti-rabbit antibody (Solarbio) and anti-mouse antibody (Solarbio) were used. Antigen–antibody complexes were visualized with ECL solution (GenDEPOT). For quantitative analysis, immunoblotting band densities were measured by image J (Kim et al., 2021).

### Stereological Cell Counts

To determine the number of BrdU- and TH-positive cells in the granule cell layer of mouse brains, an average of five sections per mouse were counted, and each experimental group consisted of five mice. BrdU-positive cells were counted in the lateral wall of the SVZ and rostral migratory stream. TH-positive cells were counted in the SN region. Unbiased stereological estimations of the total number of stained cells in each region were made using an optical fractionator. This sampling technique is not affected by tissue volume changes and does not require reference volume determinations. The sections used for counting covered the entire SN from the rostral tip of the pars compacta back to the caudal end of the pars reticulate and the lateral wall of the SVZ including RMS. This generally yielded five sections in a series. A counting frame (60%, 36,650 μm^2^) was randomly placed onto the first counting area and systematically moved through all counting areas until the entire delineated area was complete. Actual counting was performed using a 40 x object. The total number of stained cells was calculated according to the optical fractionator formula.

### Statistical Analysis

After verification of normality distribution, mean differences between experimental groups were determined by one-way analysis of variance (ANOVA) followed by a Bonferroni *post-hoc* test. Differences were considered statistically significant at *p* < 0.05. Statistical analysis was performed using the software SPSS v. 25 (Yonsei University, Seoul, Korea).

## Results

### Uric Acid-Elevating Therapy Increased Neurogenesis in the Subventricular Zone of MPTP-Induced Parkinson’s Disease Animal Model

**FIGURE 1:**
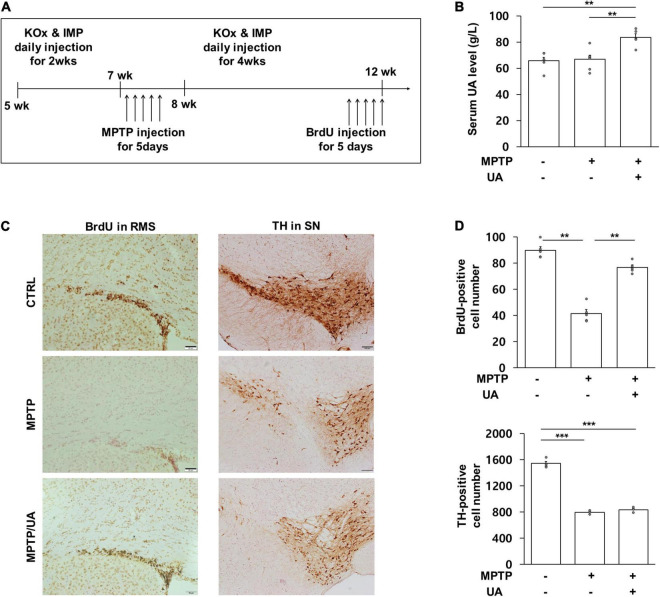
UA elevation increased the number of BrdU-positive cells in the SVZ of a MPTP-induced PD animal model. **(A)** Animal experimental schedule design. **(B)** Serum UA levels were significantly higher in mice that received IMP with KOx as compared to control and MPTP-treated mice (*n* = 8 per group). **(C,D)** The number of BrdU-positive cells in SVZ at 12 weeks. PD mice with high serum UA levels showed considerably more BrdU-positive cells as compared to PD mice with normal serum UA levels (*n* = 5 per group). The number of TH-positive cells in the SN at 12 weeks. Both PD mice with high and normal UA levels revealed a significant reduction in the number of TH-positive cells compared to the control group. There was no statistically significant difference between PD mice with high serum UA levels and those with normal levels (*n* = 5 per group). Differences among the conditions were evaluated by ANOVA with a Bonferroni correction for multiple comparison. The data are presented as mean ± SE. **p < 0.01 and ***p < 0.001.

To determine the effect UA elevation on neurogenesis in an MPTP-induced parkinsonian model, the mice were treated with mixture of KOx and IMP before MPTP treatment *via* the intraperitoneal route. The *in vivo* study design is illustrated in Figure 1A. Two weeks of KOx injection (500 mg/kg) slightly but not significantly raised the level of serum UA (88.3 g/L vs. 85.8 g/L). However, 2 weeks of co-treatment of IMP (500 mg/kg) with KOx (500 mg/kg) led to a significant increase in the level of UA (76.4 g/L) relative to baseline (63.1 g/L, *p* = 0.006, Figure 1B). To investigate whether UA-elevating therapy modulated neurogenesis, new born cells in the SVZ of MPTP-treated mice received IMP with KOx were immunostained with BrdU, a commonly used marker for neurogenesis (Kuhn et al., 2016). Compared to the control group, the number of BrdU-positive cells in ependymal and rostral migratory stream (RMS) was significantly reduced in the MPTP-treated mice by 46.2% (*p* = 0.001, Figures 1C,D). Compared to MPTP-only-treated mice, PD mice received co-treatment of IMP with KOx exhibited a significant increase in the number of BrdU-positive cells, restoring up to 85.3% (*p* = 0.006, Figures 1C,D). However, only post injection of IMP with KOx in MPTP-treated mice did not increase the number of BrdU-positive cells (Supplementary Figure 1). Next, as dopaminergic neurons can modulate neurogenic activity *via* dopaminergic receptors in the SVZ, we determined whether UA elevation in MPTP-treated mice would lead to a difference in the density of nigral dopaminergic neurons. As expected, the number of TH (tyrosine hydroxylase)-positive cells in the SN was reduced to 51.5% in MPTP-treated animals compared to the control group (*p* < 0.01, Figures 1C,D). However, the number of TH-positive cells in PD mice co-treated with IMP and KOx was not statistically different from the MPTP-only-treated group. In addition, we investigated neurogenesis in hippocampal dentate gyrus (DG). MPTP induced PD mice showed decreased number of BrdU positive cells in DG by 19.1% compared to the control group (*p* < 0.001). Compared to MPTP-treated mice, PD mice received UA elevating therapy showed significantly increased number of BrdU-positive cells (*p* < 0.001, Supplementary Figures 2A,B). These data indicate that UA-elevating therapy promotes neurogenic activity in MPTP-induced parkinsonian animals without modulating the nigrostriatal dopaminergic system.

### Uric Acid Treatment Exerted a Neuroprotective Effect and Enhanced Proliferation Against MPP^+^ in Primary Cultured Neural Precursor Cells From the Subventricular Zone

**FIGURE 2:**
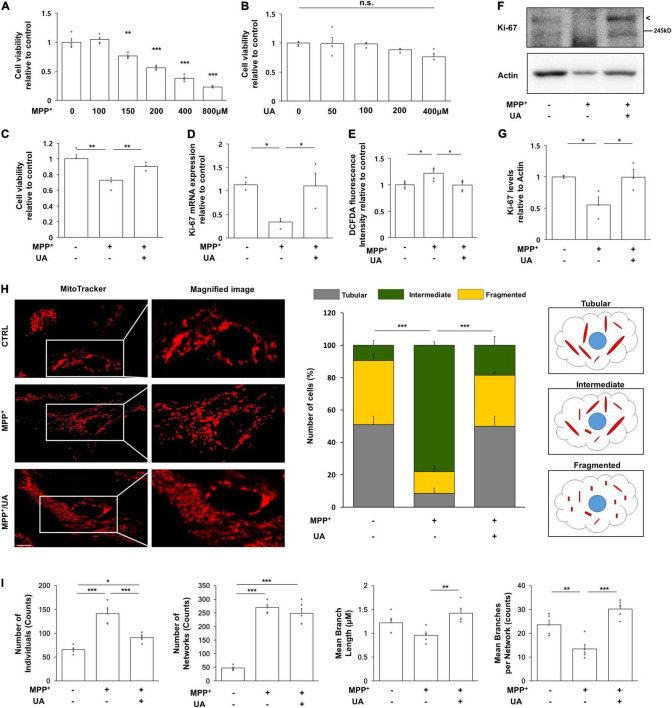
UA treatment exerted neuroprotective effect and promoted proliferation in primary cultured NPCs. **(A)** MTS analysis in MPP^+^-treated NPCs with a concentration of 100, 150, 200, 400, and 800 μM. 150 μM of MPP^+^ treatment induced about 56% of viability on NPCs (*n* = 4 per group). **(B)** MTS analysis in UA-treated NPCs with a concentration of 50, 100, 200, and 400 μM. UA treatment did not induce statistically significant cell death up to 400 μM (*n* = 4 per group). **(C)** MTS analysis in MPP^+^ treated or MPP^+^ with UA-treated NPCs at a 150 μM concentration of UA for 24 h and MPP^+^ for 72 h with UA co-treatment. UA exerted a neuroprotective effect on NPCs by restoring cell viability up to 90% compared to control (*n* = 4 per group). **(D)** Quantitative RT-PCR for proliferation marker, Ki-67. The expression level of Ki-67 was reduced to 41.6%; however, UA restored it to 92.2% compared to control. **(E)** Intracellular ROS as measured by DCFDA assay after MPP^+^ and UA treatment. MPP^+^-induced ROS was scavenged by UA pre-treatment. **(F)** Western blot for proliferation marker, Ki67 in MPP^+^ and UA-treated NPCs. **(G)** Quantification graph of Ki-67 western blot analysis. **(H)** Immunofluorescent labeling for mitotracker. MPP^+^-treated NPCs showed fragmented mitochondrial morphology, whereas UA treatment restored them similar to those of controls. **(I)** Mitochondrial network analysis using MiNA. UA treated NPCs showed lower number of individuals (puncta), longer branch length, and lower branch number per network compared to the MPP^+^ treatment group. Each five images per group were analyzed. Scale bar: 10 μm. Differences among the conditions were evaluated by ANOVA with a Bonferroni correction for multiple comparison. The data are presented as the mean ± SE. *p < 0.05, **p < 0.01, and ***p < 0.001.

Primary cultured NPCs were positive for NES (nestin), SOX2 (SRY-box transcription factor2), DCX (doublecortin), and MSI1 (musashi RNA binding protein 1), but negative for GFAP (glial fibrillary acidic protein) (Supplementary Figure 3A). To determine the purity of cultured NPCs, cells were stained with NES and GFAP. All DAPI positive cells were co-localized with NES, but not GFAP and NeuN (Supplementary Figure 3B). To examine the effect of UA on cell survival followed by MPP^+^ treatment, primary cultured NPCs were treated with MPP^+^ at various concentrations for 72 h. MPP^+^ treatment with a concentration of 150 μM or more markedly decreased cell viability of NPCs in a dose-dependent manner (Figure 2A). However, UA treatment at various concentrations did not result in decreased viability of NPCs (Figure 2B). Next, we examined if UA treatment exhibited a neuroprotective effect against MPP^+^-induced cytotoxicity in NPCs. Similar to *in vivo* data, UA treatment in MPP^+^-treated NPCs exerted a prosurvival effect, restoring cell viability up to 90.1% compared to the MPP^+^-treated group (*p* = 0.049, Figure 2C). On evaluating proliferation of NPCs, the mRNA expression level of Ki-67 was significantly decreased in MPP^+^-treated NPCs, whereas UA treatment markedly increased the level of Ki-67 relative to the MPP^+^ treatment group (*p* = 0.026, Figure 2D). Similarly, UA treatment significantly increased the protein level of Ki-67 compared to the MPP^+^ treatment group (Figures 2F,G). To determine that detected Ki-67 in western blot and real-time PCR is solely from NPCs, we stained NPCs with NES and Ki-67. The immunofluorescence staining data shows that Ki-67 is co-localized with NES (Supplementary Figure 4). We also measured intracellular ROS level in NPCs. As expected, MPP^+^ treatment increased ROS level compared to controls (*p* = 0.023, Figure 2E). However, when UA was treated prior to MPP^+^ treatment for 24 h, it significantly scavenged excessive ROS, restoring back to control level (*p* = 0.047, Figure 2E). These data suggest that UA not only increased the survival of NPCs against MPP^+^ toxicity, but also enhanced proliferation *in vitro.* Next, the modulatory mechanism of UA on neurogenic activity was further studied in mitochondrial dynamics. To analyze mitochondrial morphology, we performed immunofluorescence staining using mitotracker. MPP^+^-treated NPCs displayed many more fragmented mitochondria over 78% rather than tubular shape. On contrast, UA treated NPCs exhibited more interconnected, elongated and filamentous mitochondria similar to those of naïve NPCs. About 18% of UA treated NPCs showed fragmented mitochondria (Figure 2H). We also confirmed the effect of UA on mitochondrial network morphology using MiNA. UA treated NPCs showed lower number of individuals (puncta), longer branch length, and lower branch number per network compared to the MPP+ treatment group (Figure 2I).

### Uric Acid Treatment Rescued the Abnormal Mitochondrial Phenotype Observed in Primary Cultured Neural Precursor Cells by Modulating Mitochondrial Dynamics

**FIGURE 3:**
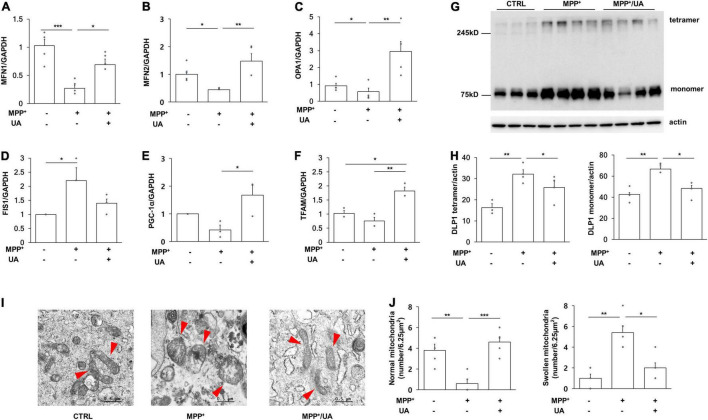
UA treatment rescued the mitochondrial phenotype *via* modulating mitochondrial dynamics in NPCs. **(A)** Quantitative RT-PCR for mitochondrial fusion marker, MFN1. **(B)** Quantitative RT-PCR for mitochondrial fusion marker, MFN2. **(C)** Quantitative RT-PCR for mitochondrial fission marker, OPA1. **(D)** Quantitative RT-PCR for mitochondrial fission marker, FIS1. **(E)** Quantitative RT-PCR for mitochondria master regulator, PGC-1α. **(F)** Quantitative RT-PCR for mitochondrial transcription factor TFAM. MPP^+^ treatment led to a significant decrease in the expression level of MFN1, MFN2, PGC-1α, and TFAM and UA treatment has restored it. On the contrary, the expression level of FIS1 and OPA1 was increased when exposed to MPP^+^-, and UA treatment restored it (*n* = 5 per group). **(G,H)** Western blot for mitochondrial fission regulator Dlp1 tetramer and monomer form. Both tetramer and monomer form were increased with MPP^+^ treatment, which was rescued by UA (*n* = 3 for CTRL group, *n* = 4 for MPP^+^ and MPP^+^/UA group). **(I)** TEM analysis revealed that MPP^+^ treatment induced severe abnormal morphology of mitochondria with a swollen body and fragmented phenotype. However, UA treatment returned mitochondria morphology to a similar state as control NPCs. The arrows indicate mitochondria. **(J)** MPP^+^ treated NPCs showed reduced normal mitochondria and increased swollen mitochondria, however, UA treatment rescued it. Differences among the conditions were evaluated by ANOVA with a Bonferroni correction for multiple comparison. The data are presented as mean ± SE. *p < 0.05, **p < 0.01, and ***p < 0.001.

To investigate the mitochondrial dynamics modulatory mechanism in detail, mitochondrial dynamics-related genes were measured with quantitative real-time PCR analysis. The expression level of mitochondrial fusion marker, MFN1 (mitofusin 1), MFN2 (mitofusin 2), and OPA1 (mitochondrial dynamin like GTPase) was significantly decreased in NPCs following MPP^+^ treatment as compared to the control group (*p* = 0.000, *p* = 0.046, *p* = 0.048), whereas UA treatment restored them in MPP^+^-treated NPCs (*p* = 0.023, *p* = 0.01, *p* = 0.002, Figures 3A–C). Conversely, expression level of FIS1 (mitochondrial fission 1) was markedly increased in NPCs approximately two- and three-fold following MPP^+^ treatment, repectively (*p* = 0.021); however, UA treatment counteracted them in MPP^+^-treated NPCs (Figure 3D). Under MPP^+^ treatment, mitochondrial master regulator, PGC-1α (PPARG coactivator 1 alpha), tended to decrease in NPCs but did not reach statistical significance (*p* = 0.406), whereas UA treatment significantly upregulated the expression of PGC-1α compared to the only-MPP^+^-treated group (*p* = 0.029, Figure 3E). In addition, UA treatment in MPP^+^-treated NPCs significantly increased the expression of mitochondrial transcription factor, TFAM (transcription factor A, mitochondrial) (*p* = 0.001) relative to the MPP^+^-treated group (*p* = 0.001, Figure 3F). Additionally, we measured protein level of DLP1 (GTPase Dynamin-related protein 1) tetramer and monomer form to investigate whether Dlp1 was hyperactivated. Both tetramer and monomer form of DLP1 was increased in MPP^+^ treated NPCs (*p* = 0.007, *p* = 0.006), while UA treatment counteracted it (*p* = 0.043, *p* = 0.019, Figures 3G,H). These data indicate that UA has scavenged MPP^+^ toxins by modulating mitochondrial dynamics. Next, we evaluated the changes of actual mitochondrial morphology using TEM analysis. Mitochondrial morphology was preserved in control NPCs, with well-formed cristae and undisrupted mitochondria membranes. However, mitochondria following MPP^+^ treatment were filled with discontinuous cristae, electron-dense structures, and damaged double-membranes. Additionally, mitochondria in MPP^+^-treated NPCs exhibited swollen bodies (Figure 3I). The number of normal and swollen mitochondria was counted in restricted area and presented in graph (Figure 3J). These data suggest that treatment of MPP^+^ leads to mitochondrial damage, possibly triggering fission process and/or inhibiting fusion process. However, UA treatment successfully returned mitochondrial morphology to a healthy form, similar to those of naïve NPCs, through a possible rescue of mitochondrial dynamics balance of fusion and fission processes.

### Parkinson’s Disease Mice With Elevated Serum Uric Acid Levels Showed Increased BrdU-Positive Cell Numbers Through Modulation of Mitochondrial Dynamics Key Actors

**FIGURE 4:**
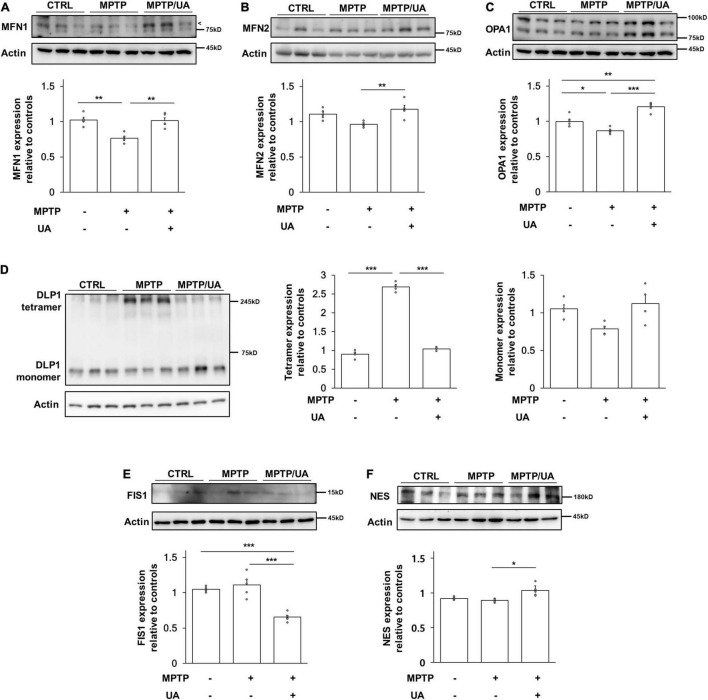
PD mice with high serum UA level showed modulated mitochondrial dynamics. **(A)** Western blot for mitochondrial fusion marker, MFN1. **(B)** Western blot for mitochondrial fusion marker, MFN2. **(C)** Western blot for mitochondrial fusion marker, OPA1. The expression levels of MFN1, MFN2, and OPA1 were decreased in PD mice and upregulated in UA-elevated PD mice (*n* = 5 per group). **(D)** Western blot for mitochondrial fission regulator Dlp1 tetramer and monomer form. **(E)** Western blot for mitochondrial fission marker, FIS1. The expression level of Dlp1 tetramer form and FIS1 were increased in PD mice and significantly suppressed in PD mice with high serum UA levels (*n* = 5 per group). **(F)** Western blot for neural stem cell marker, NES. The expression levels of NES were significantly increased in UA-elevated PD mice compared to control or PD mice with normal UA levels (*n* = 5 per group). Differences among the conditions were evaluated by ANOVA with a Bonferroni correction for multiple comparison. The data are presented as mean ± SE. *p < 0.05, **p < 0.01, and ***p < 0.001.

Next, mitochondrial dynamics-related proteins were analyzed to investigate whether UA elevation could modulate mitochondrial dynamics in the SVZ of MPTP-treated animals. The protein expression of mitochondrial fusion marker, MFN1 (mitofusin 1), was significantly decreased in MPTP-treated animals as compared to control mice (*p* = 0.001, Figure 4A), and another marker, MFN2, was slightly decreased in the parkinsonian model as compared to healthy mice (*p* = 0.054, Figure 4B). The expression of the OPA1 (OPA1 mitochondrial dynamin like GTPase) tended to decrease in MPTP-treated mice relative to control mice (*p* = 0.031, Figure 4C). However, UA elevation in MPTP-treated animals led to an increase in the expression of MFN1 (*p* = 0.001), MFN2 (*p* = 0.005), and OPA1 (*p* = 0.000) as compared to the MPTP-only-treated group (Figures 4A–C). To determine whether DLP1 (GTPase Dynamin-related protein 1), mitochondrial fission-related protein, was activated, we measured the expression level of DLP1 tetramer form. The level of DLP1 tetramer form was significantly increased in MPTP-treated animals compared to control animals, which was down-regulated after UA elevating therapy (*p* = 0.000, Figure 4D). However, there was no significant difference in monomer form among three groups. Another fission regulator, FIS1, was down-regulated with UA-elevating therapy compared to the control and MPTP groups (*p* = 0.000, Figure 4E). Meanwhile, UA elevation in MPTP-treated animals led to a significant increase in expression of Dlp1 and FIS1 (*p* = 0.029 and 0.000, respectively) compared to the MPTP-only-treated group (Figures 4D,E). Finally, UA elevation led to a significant increase in expression of NES in the SVZ of MPTP-treated animals (*p* = 0.021, Figure 4F).

## Discussion

This study aimed to investigate whether elevation of serum UA levels modulate neurogenic activity in a parkinsonian model. The major findings of present study are (1) UA elevation in MPTP-induced PD mice led to an increased number of BrdU-positive cells in the SVZ including RMS and DG compared to MPTP-only-treated mice. (2) UA treatment exerted a prosurvival effect, restoring viability and proliferation of NPCs against MPP^+^ treatment. (3) In the process of modulating neurogenesis, our *in vitro and in vivo* data demonstrated that UA elevation regulated mitochondrial dynamics *via* promoting fusion machinery. The results suggest that UA enhances neurogenic activity in the SVZ of a parkinsonian model by modulating mitochondrial dynamics.

Although the underlying molecular mechanisms of the altered neurogenesis in PD remain unknown, neurochemical deficits of dopamine (Mishra et al., 2019b), indirect effects of growth factor release, and α-synuclein accumulation in NPCs of the SVZ *via* the disease process may influence neurogenic activity (Winner et al., 2012). Several studies have demonstrated the effect of α-synuclein in the hippocampus and SVZ of transgenic animal models and murine embryonic stem cells (Winner et al., 2004, 2008; Crews et al., 2008; Kohl et al., 2012). In addition, recent evidence has demonstrated that mitochondrial dysfunction and inappropriate regulation of mitochondrial dynamics have detrimental effects on neurogenesis in the embryonic as well as adult brain. During neurogenesis, mitochondrial dysfunction resulting from deletion of the mitochondrial oxidoreductase protein AIF within the early NSC population disrupted the neurogenic pathway completely, including self-renewal capacity of NSC and proliferation and differentiation of NPCs (Khacho et al., 2017). Likewise, disruption of mitochondrial function by loss of the mitochondrial transcription factor, TFAM, when restricted to the adult NSC population, leads to impairment in adult neurogenesis. During embryonic neurogenesis, mitochondrial dynamics seem to play an important role in the stem cell decision-making process enhanced mitochondrial fusion promotes NSC self-renewal, while mitochondrial fragmentation initiates the commitment of NSC to neuronal differentiation and neuronal maturation (Khacho and Slack, 2018). A recent study reported that inhibition of mitochondrial fission improved mitochondrial dynamics and stimulated hippocampal neurogenesis in Down syndrome animal model (Valenti et al., 2017). Therefore, maintenance of proper mitochondrial dynamics would be an important strategy to modulate neurogenic activity in PD.

The present study has demonstrated that UA elevation in MPTP-induced PD mice led to an increased number of BrdU-positive cells in the SVZ compared to MPTP- only-treated mice, restoring up to 85% of neurogenic activity compared to the control mice. In a cellular model using primary culture of NPCs, UA treatment exerted a prosurvival effect, restoring cell viability of NPCs against MPP^+^ treatment and led to increased proliferation markers. As impairment in neurogenesis is known to be a critical factor in neurodegenerative diseases including PD, modulating it could be an effective strategy in disease-modifying treatment. Accumulating clinical data have also reported an association between high serum UA levels and lower likelihood of developing PD (de Lau et al., 2005). Furthermore, we found that the hippocampal neurogenesis was also enhanced with UA-elevating therapy. Previous studies have demonstrated that UA decreases the risk of dementia in general population mice (Euser et al., 2009) and improves cognitive performance in PD mice through the Nrf2-ARE signaling pathway (Huang et al., 2017). Hence, it is possible that UA could improve cognitive function through modulation of hippocampal neurogenesis. A further research is warranted to investigate whether UA would be helpful in AD models by modulating hippocampal neurogenic activity.

Interestingly, we have demonstrated that UA may modulate neurogenesis by remodeling mitochondria. Mitochondria are dynamic organelles that constantly change their size and morphology in response to the environment in the machinery of fission and fusion. In mammals, MFN1, MFN2, and OPA1 are responsible for mitochondrial fusion, while Dlp1 and FIS1 are required for mitochondrial fission. Fission is a process of breaking apart into smaller fragments, which is important for segregating dysfunctional mitochondria from healthy counterparts (Ni et al., 2015). Therefore, this process plays an important role in quality control of maintaining healthy mitochondria, further sustaining healthy neurons that require considerable energy. In the process of neurogenesis, a critical amount of ATP is required to facilitate cytoskeletal rearrangement, neuronal sprouting, and organelle transport. Being a primary source of cellular ATP, healthy mitochondrial function is necessary for the brain to perform neurogenesis effectively. We also demonstrated that UA up-regulated mitochondrial biogenesis-related genes including PGC-1α and TFAM. According to previous study, PGC-1α guarantees the production of healthy mitochondria and a correct equilibrium between mitophagy and mitochondrial biogenesis, thus blocking excessive ROS (Baldelli et al., 2014). In terms of the association between oxidative stress and neurogenesis, several studies have highlighted the fact that oxidative stress load directly affects cellular states by modulating the redox state of NSCs or NPCs, given that adult neurogenesis is a high-energy-consuming process, and thus, leads to ROS accumulation (Yuan et al., 2015). MPTP, the neurotoxin widely known to induce PD models, is highly lipophilic, and so it can rapidly cross the blood brain barrier after systemic administration. MPTP is metabolized within the brain to active toxin MPP^+^ by monoamine oxidase, resulting in mitochondrial complex I dysfunction and impaired mitochondrial homeostasis (Kopin, 1987). Mitochondrial malfunction, already problematic in the process of PD, makes stem cells vulnerable to oxidative stress, which in turn accelerates impairment of neurogenesis. Moreover, emerging data from human and animal models of PD have reported that α-synuclein has an important role in the control of neuronal mitochondrial dynamic processes (Banerjee et al., 2010; Melo et al., 2018), suggesting a strong candidate for a pathogenic mechanism underpinning PD pathogenesis beyond mitochondrial biogenesis, a well-known factor. Therefore, UA, having potent antioxidant properties, would defend mitochondrial homeostasis and dynamics against MPTP-induced oxidative stress, which can lead to maintenance of neurogenic activity in the SVZ of PD models. There are several other neurotoxins inducing PD experimental model. Rotenone is reported to cause excessive mitochondrial fission, resulting disrupted mitochondrial dynamics (Peng et al., 2016) and 6-hydroxydopamine (6-OHDA) promoted mitochondrial fission process, elevating Drp-1 level (Galindo et al., 2012). Also rats who received surgical injection of human alpha-synuclein showed increased mitochondrial fission (Park et al., 2020). As our study described mitochondrial dynamics regulation as a mechanism underlying enhanced neurogenesis, it is plausible that UA could work in other PD models as well. Given that the precise mechanism of mitochondrial dynamics by UA is still elusive, future study focusing on down signaling pathways of UA-associated mitochondrial remodeling is warranted. Accordingly, our data provide additional evidence that modulation of neurogenic activity *via* modulating mitochondrial dynamics by a UA-elevating strategy may be an intriguing mechanism in the development of disease modifying treatment of PD.

Recent studies have suggested the important role of dopaminergic modulation in the neurogenic activity of the SVZ by demonstrating that NPCs in the SVZ express dopaminergic receptors and dopaminergic innervation from the SN in a 6-OHDA-induced PD rat model (Hoglinger et al., 2004; Mishra et al., 2019a). Specifically, dopamine agonists augment neurogenesis in animal models of PD (Winner et al., 2009), and chronic use of levodopa has a positive effect on the number of NSCs in the SVZ of patients with PD (O’Sullivan et al., 2011). Based on these previous data, the present study evaluated the density of nigral dopaminergic neurons between MPTP-induced PD animals with and without UA-elevating therapy to exclude the direct effect of nigral dopamine on neurogenesis in the SVZ. However, there was no significant change in nigral dopaminergic density between groups, suggesting that the modulating effect of UA on neurogenesis in NPCs of the SVZ may reflect a primary effect of UA itself rather than an indirect effect of dopaminergic medication.

This study has several limitations to extend clinical implications. First, although neuroprotective effect of UA in PD incidence and progression seems to be sex-dependent (O’Reilly et al., 2010), the present study examined effect of UA elevation on neurogenic activity only in male PD mice. Further study of UA-mediated neurogenic activity in female animals is also necessary. Second, although serum UA levels increased following injection of IMP with KOx, post-treatment after MPTP induction without pre-treatment prior to MPTP induction did not lead to a significant increase in neurogenesis. In both an animal and cellular model, pre-treatment of UA enhanced neurogenesis and exerted prosurvival effect of NPCs. Regarding these data, neurogenic modulation of UA seems to be effective in the premotor stage of PD; however, this issue should be investigated in future studies. Third, we could not provide mitochondrial morphology in animal tissue, which may limit the role of UA in mitochondrial dynamics. Finally, when UA is elevated beyond the normal range, it increases risk of gout and cardiovascular diseases. This issue requires further study to determine the appropriate UA level exerting beneficial effects in parkinsonian disorder.

## Conclusion

In summary, this study demonstrates that UA elevation can enhance neurogenic activity in the NPCs of the SVZ in parkinsonian animal and cellular models by modulating mitochondrial dynamics.

## Data Availability Statement

The original contributions presented in the study are included in the article/Supplementary Material, further inquiries can be directed to the corresponding author.

## Ethics Statement

All procedures were performed in accordance with the Laboratory Animals Welfare Act, the Guide for the Care and Use of Laboratory Animals and the Guidelines and Policies. The rodent experiment was approved by IACUC (Institutional Animal Care and Use Committee) in the Yonsei University Health System. ARRIVE guidelines were followed (http://arriveguidelines.org).

## Author Contributions

JL contributed to study design, draft, performing experiments, and revise the manuscript and figures. YS, YK, HK, and DK contributed to analysis of data, verifying the underlying data, and interpretation of results. SC and HY contributed to interpretation of results. JS contributed to conception, study design, analysis of data, and interpretation of results. PL contributed to conception, study design, revise the manuscript and figures, supervision, and funding acquisition. All authors edited and approved the manuscript.

## Conflict of Interest

The authors declare that the research was conducted in the absence of any commercial or financial relationships that could be construed as a potential conflict of interest.

## Publisher’s Note

All claims expressed in this article are solely those of the authors and do not necessarily represent those of their affiliated organizations, or those of the publisher, the editors and the reviewers. Any product that may be evaluated in this article, or claim that may be made by its manufacturer, is not guaranteed or endorsed by the publisher.

## Footnotes

1
http://arriveguidelines.org
