# Supplementary material for: Uric Acid Enhances Neurogenesis in a Parkinsonian Model by Remodeling Mitochondria
Source: Front Aging Neurosci. 2022 Jun 2;14:851711. doi: 10.3389/fnagi.2022.851711 (PMC9201452; doi:10.3389/fnagi.2022.851711)

Supplementary Figure 1. Post only injection of IMP with KOx failed to increase the number of BrdU-positive cells in RMS of a MPTP-induced PD animal model. (A): BrdU-DAB staining in RMS of SVZ at 12 weeks. Post only injection of IMP with KOx did not significantly modulate the number of BrdU-positive cells in parkinsonian model. Pre- and post-treatment of IMP with KOx significantly increased the number of BrdU-positive cells compared to MPTP-induced PD animals. Scale bar: 100μm

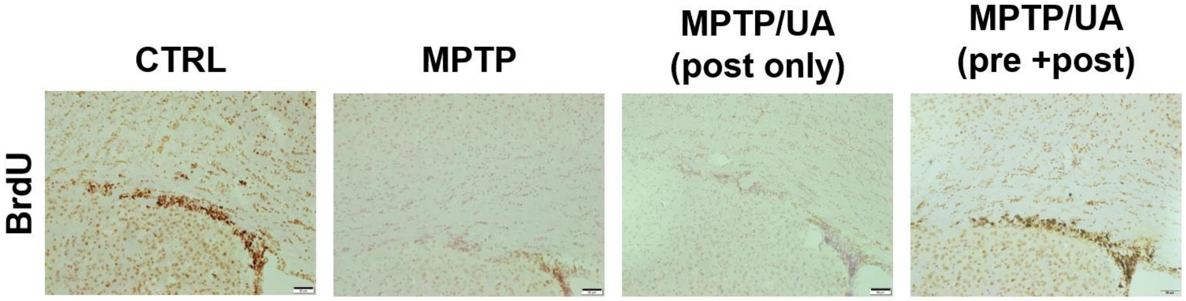

Supplementary Figure 2. UA elevation increased the number of BrdU-positive cells in the hippocampal DG of a MPTP-induced PD animal model. (A): BrdU-DAB staining in DG at 12 weeks. PD mice with high serum UA levels showed markedly increased BrdU-positive cells compared to PD mice with normal UA levels. (B): Quantification of BrdU-positive cells. There was statistically significant difference between PD mice with high serum levels and those with normal levels (n = 6 per group). The data are presented as mean ± SE. $^{***}p<0.001$. Scale bar: 100µm

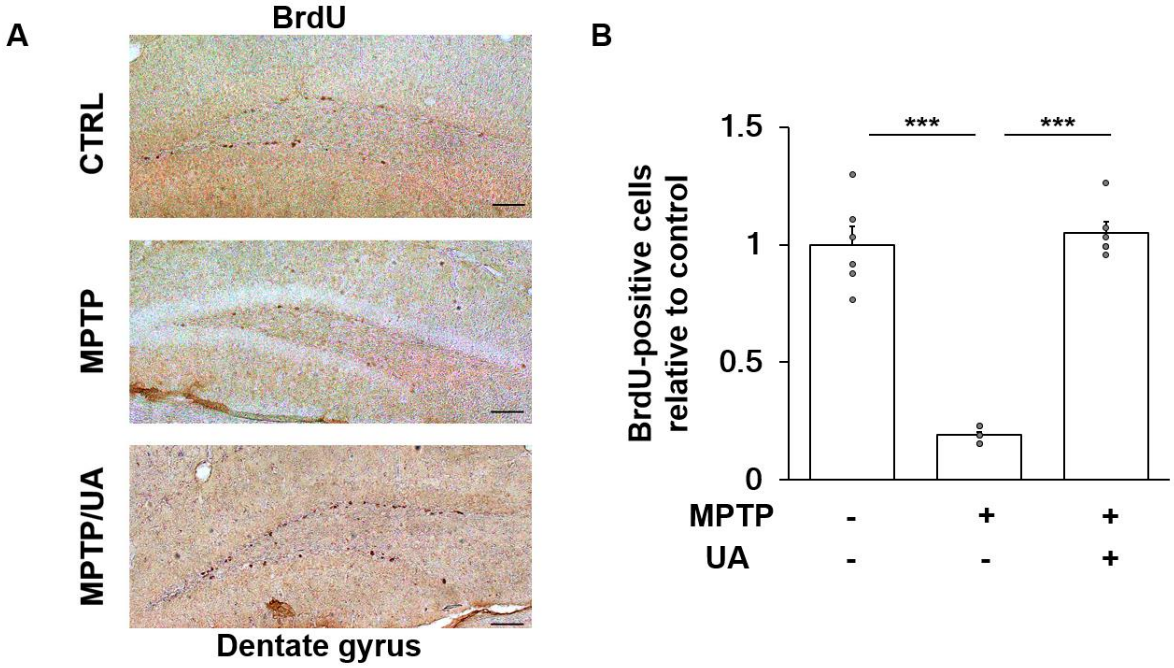

Supplementary Figure 3. Characterization and purity of primary cultured NPCs in SVZ. (A): Primary cultured NPCs were positive for NES, SOX2, DCX, and MSI1, but negative for GFAP. The identified NPCs were further used for evaluation. (B): To determine the purity of cultured NPCs, cells were stained with Nestin, GFAP, and NeuN. All DAPI positive cells express Nestin, but no GFAP and NeuN. Scale bar: 20μm

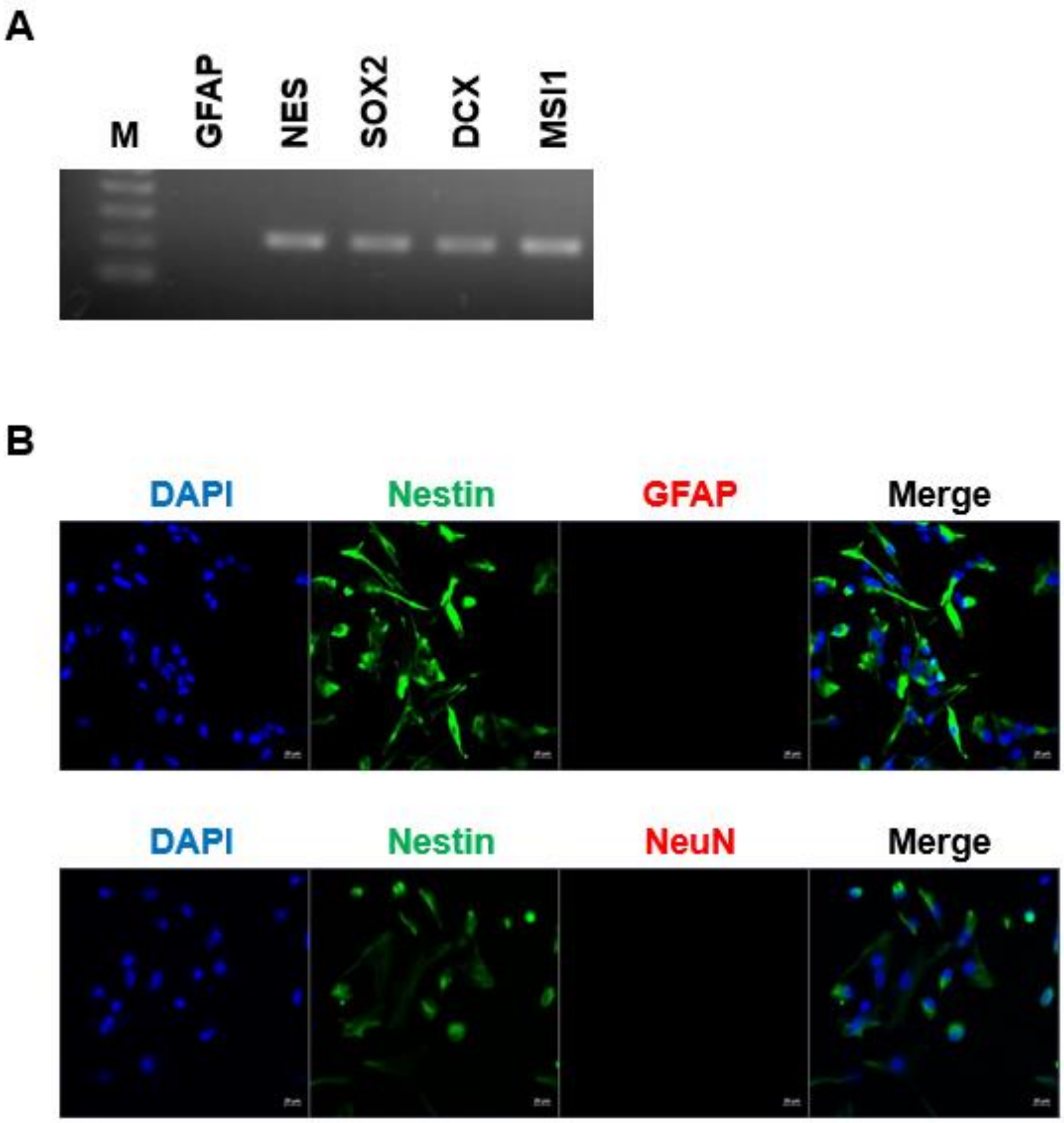

Supplementary Figure 4. Double immunofluorescent staining of Nestin and Ki-67 in primary cultured NPCs. (A): Ki-67 is co-localized with Nestin. Scale bar: 20μm

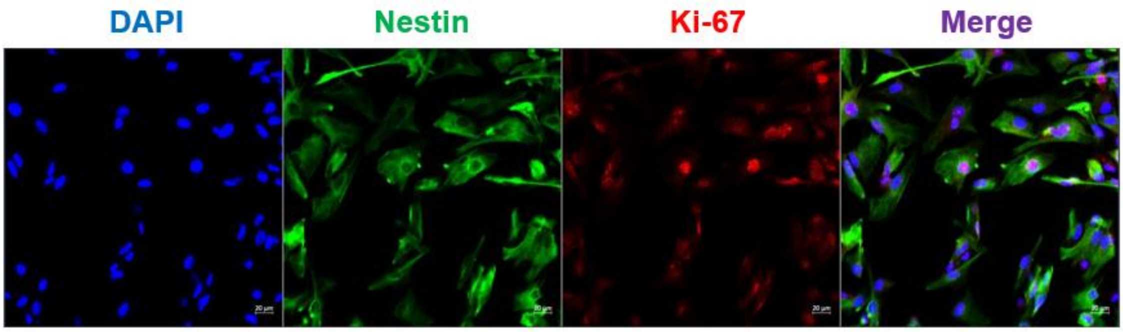

Supplement: Supplementary file 1 [file Data_Sheet_1.pdf]
